# Breathing Uneasy: Fit-Tested N95 Respirator Access in Washington State Long-Term Care Facilities

**DOI:** 10.1093/geroni/igab046.603

**Published:** 2021-12-17

**Authors:** Carolyn Ham, Mikkie Nakamura

**Affiliations:** 1 Washington State Department of Health, Olympia, Washington, United States; 2 Washington State Department of Health, Shoreline, Washington, United States

## Abstract

Long-term care facilities (LTCF) have been disproportionately impacted by illness and death from COVID-19. Shortages of respirators for staff, especially Particulate Filtering Facepiece Respirators (N95), have limited LTCFs ability to follow public health recommendations for preventing COVID-19 transmission. Use of N95 respirators was infrequent in Washington State (WA) LTCFs prior to May 2020. N95 respirators must be individually fit tested to provide intended protection; a fit test is a procedure that tests the seal between the N95 respirator and the wearer’s face. The WA Department of Health (WA DOH), collaborated with stakeholders to survey LTCFs in November 2020 regarding needs for fit tested respirators and analyzed responses (n=384). Responses by facility type: 8.3% nursing homes, 17.7% assisted living, 62.8% adult family home, 11.2% other. In WA, adult family homes (AFH) are licensed for six or fewer residents. 23.70% of LTCFs indicated they did not have any N95 respirators in stock at their facility; 96.7% of these were AFH. In August 2020 WA DOH surveyed AFH owners and received 110 responses; 9.76% reported having at least one staff member fit tested for respirators. Smaller facilities may experience increased burden in accessing N95 respirators and fit testing due to lack of established relationships with suppliers and small volumes being purchased. WA DOH used federal COVID funding to contract with mobile fit testing providers and prioritized AFHs for this service. Between December 1, 2020-February 28, 2021, staff at 290 LTCFs were fit tested. The project will continue throughout 2021.

